# High Throughput Screening Method to Explore Protein Interactions with Nanoparticles

**DOI:** 10.1371/journal.pone.0136687

**Published:** 2015-08-27

**Authors:** Irem Nasir, Warda Fatih, Anja Svensson, Dennis Radu, Sara Linse, Celia Cabaleiro Lago, Martin Lundqvist

**Affiliations:** 1 Center for Molecular Protein Science, Department of Biochemistry and Structural Biology, Lund University, Lund, Sweden; 2 Upper 2nd school, Klippan, Sweden; Kermanshah University of Medical Sciences, ISLAMIC REPUBLIC OF IRAN

## Abstract

The interactions of biological macromolecules with nanoparticles underlie a wide variety of current and future applications in the fields of biotechnology, medicine and bioremediation. The same interactions are also responsible for mediating potential biohazards of nanomaterials. Some applications require that proteins adsorb to the nanomaterial and that the protein resists or undergoes structural rearrangements. This article presents a screening method for detecting nanoparticle-protein partners and conformational changes on time scales ranging from milliseconds to days. Mobile fluorophores are used as reporters to study the interaction between proteins and nanoparticles in a high-throughput manner in multi-well format. Furthermore, the screening method may reveal changes in colloidal stability of nanomaterials depending on the physicochemical conditions.

## Introduction

Technical, biological and biomedical applications of nanoparticles depend on the interaction between nanoparticles and biomolecules such as proteins [1–6] and lipids [6–8]. Important considerations in biological fluids with ten thousands of biomolecules competing for the nanoparticle surface are affinities, specificities and exchange rates [9–11]. The technical applicability as well as potential biohazards will depend also on the structural and functional perturbations of the interacting proteins [12, 13]. High throughput and accurate screening methods for detection of interactions between proteins and nanoparticles are therefore desirable, in fields such as bionanotechnology, nano-safety and nano-medicine. Both qualitative and quantitative screening methods are needed for identification of interacting proteins and measurements of the interaction parameters in terms of rate and equilibrium constants, conformational transitions and functional consequences. Many different analytical methods have been applied to study the interaction between a protein and a nanoparticle surface and the influence of an interaction on the native state of the protein, among others: circular dichroism [11, 14], ellipsometry [15], infrared spectroscopy [16], atomic force microscopy [17], fluorescence labelling of the protein [18], nuclear magnetic resonance [11, 19–21], analytical ultracentrifugation [11], limited proteolytic cleavage in combination with mass spectrometry [22] and dynamic light scattering coupled with ζ potential change [23].

Interactions between proteins and nanoparticles are governed by size, shape, material (and surface modification) of the particle, and the medium conditions [24, 25]. Proteins that adsorb (i.e. interact with dissociation constants below 10^−7^–10^−5^ M) [26, 27] to nanoparticles may resist or undergo structural changes [1, 10, 11, 20, 22, 28–30], which compromises functions that rely on the native structure (i.e. an enzyme may lose its catalytical ability [18, 31–33]), or may lead to the exposure of new epitopes [13, 34] that trigger unwanted biological responses *in vivo*. Methods for high throughput and non laborious screening of interaction parameters and consequences of a particular combination of protein and nanoparticle would make the design of future bionanomaterials more efficient to improve the applicability and minimizing biohazards at early stages.

## Method Outline

**Fig 1:**
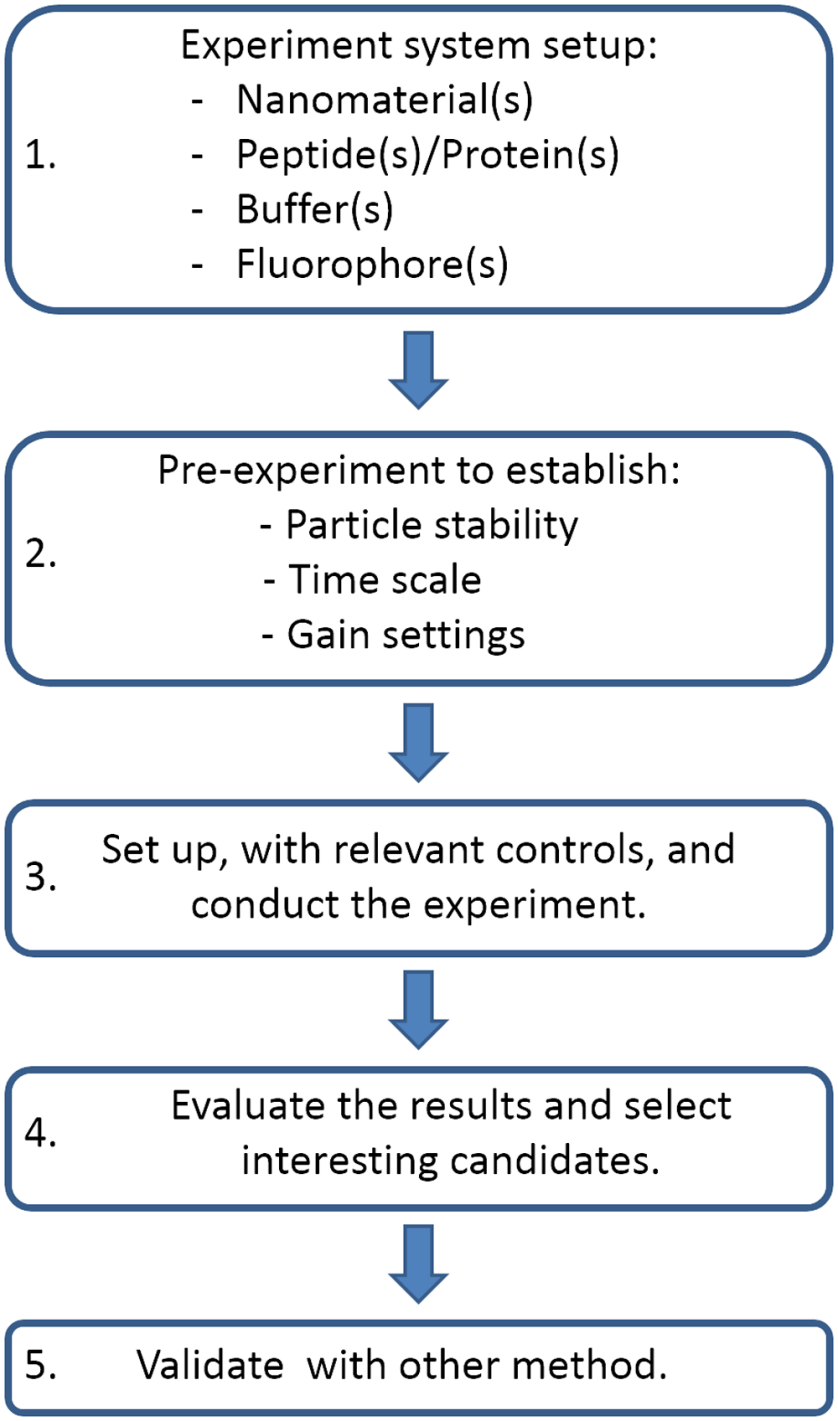
Method outline. Short description of the method.

This article presents a high throughput method to screen for protein interactions with nanoparticles. Many partners can be screened simultaneously using appropriate fluorophores and a plate reader with fluorescence detection. The kinetics of the interactions and the potential protein structural rearrangements can be followed on time scales from milliseconds to days. The screening method can also be used to study nanoparticles’ colloidal stability in different buffers, with or without proteins, over time. Fig 1 outlines the steps in the proposed screening method.

At Step 1 the system to study is selected in terms of nanoparticles, peptide/protein, buffer, and fluorophore. In principle, all fluorophores that are water soluble and undergo a significant change in emission spectrum upon interactions with different surfaces can be used with this method. For the particular purpose of this article, we have chosen two solvatochromic dyes, 8-Anilino-1-naphthalenesulfonic acid (ANS) and Nile red (NR), for which the emission intensity and maximum shift in response to changes in the polarity of the surrounding environment [35, 36]. ANS and NR have been used in numerous protein unfolding studies [35], and, ANS has been used to study the interactions between proteins and nanoparticles [14]. Hence, protein interaction/adsorption with or without subsequential unfolding event(s) can be tracked using these commercially available dyes. However, the screening method per se is not limited to these dyes. Other flourophores may be selected, for some examples see Klymchenko and Mely [36]. The plate reader has to be set up with excitation and emission filters that match the fluorophore that will be used or a monochromator that spans the excitation and emission wavelengths of the selected fluorophores.

At Step 2 a test experiment is conducted in order to optimize the experimental setup for the system under study in terms of mixing time, spacing of time points and gain. Furthermore, the colloidal stability of the nanoparticles, both in selected buffer and in presence of the dye, and their signal contribution due to fluorescence or light scattering at the selected wavelengths are important aspects to be sorted out before starting a large-scale screening experiment.

One more aspect to take into consideration is that the adsorption process and structural rearrangements can occur at different time scales [11]. If this process occurs within seconds after mixing, the screening method needs to be adapted. To be able to capture the changes that take place within milliseconds either particle or protein has to be injected into the sample well, using an automatic injector in the plate reader, to minimize the time between mixing and the start of the measurement.

To acquire the best signal to noise ratio and to avoid data overflow in Step 3, the samples from the test experiment are used to calibrate the gain. Ideally, the fluorescence intensity (I_F_) should not exceed 80–90% of the instrument limit and the gain is adjusted using the sample with the highest I_F_ after a sufficiently long incubation time (e.g. overnight) in the test experiment.

At step 3 the detailed large-scale experiment is executed with predefined parameters from step 2. Sample mixing is done in following order: buffer, fluorophore, protein and particles. This order enables a competition between protein and fluorophore to adsorb on the particle surface, if applicable. Depending on the system that is going to be studied and the criteria that are of interest, the sample mixing order can be adjusted. For example, if the fluorophore preferentially binds to the active site of a protein, it can be added last to investigate if the nanoparticle blocks the access to the active site[14]. Step 4 includes data analysis and at step 5 the interactions are confirmed using complementary methods.

## Screening Output and Analysis

**Fig 2:**
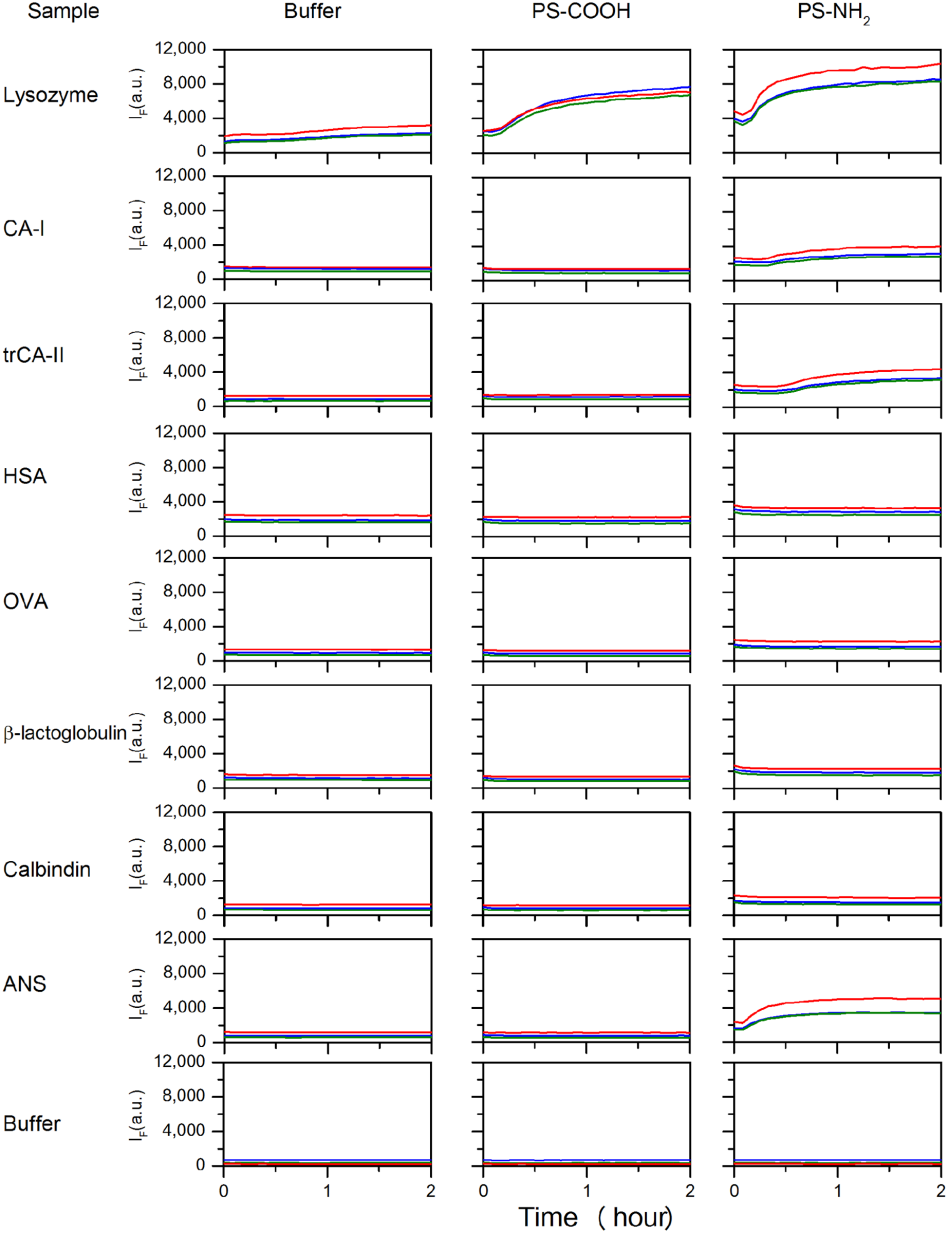
Screening raw data, generated by the plate reader. The interactions between 7 proteins and 2 particles was followed with ANS fluorescence monitored at 3 emission wavelength; blue = 460 nm, green = 475 and red = 520 nm, over time. The shown results are the average of three sample replicates.

**Fig 3:**
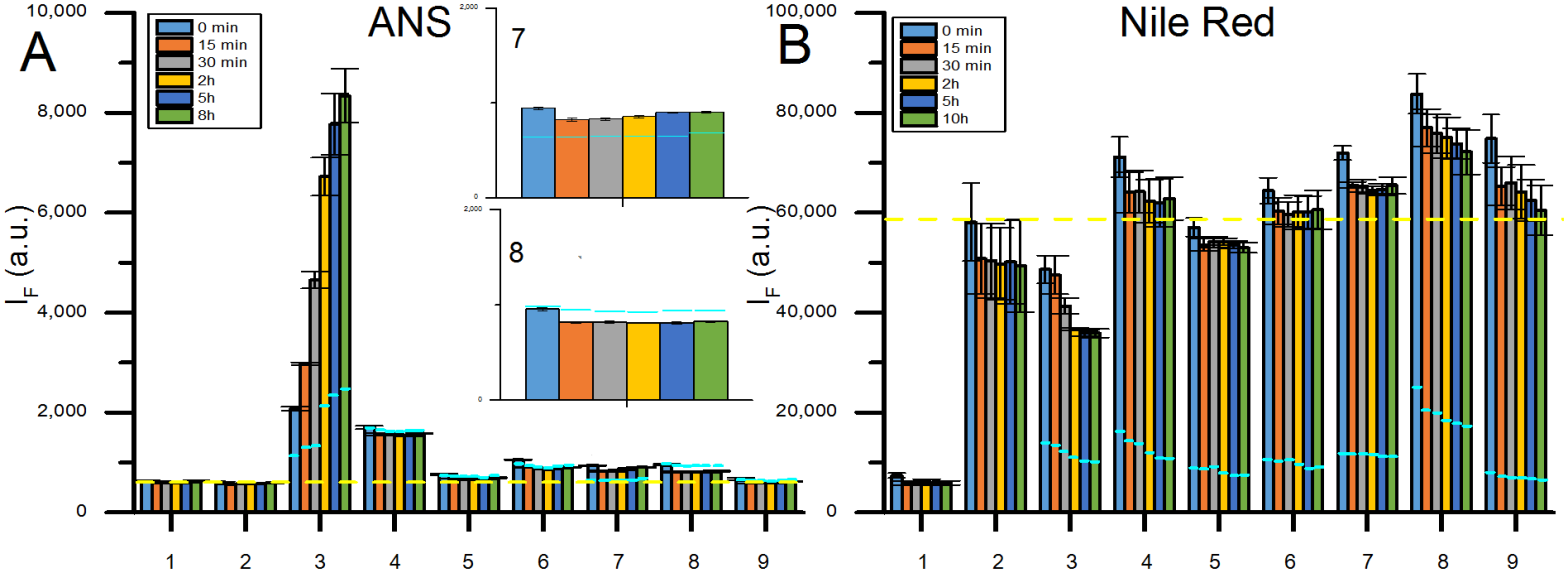
Screening results for 7 proteins and PS-COOH, ANS data in panel A and NR data in panel B, with corresponding controls. Bars represent the mean intensity value from three individual samples, with standard deviation in corresponding error bars, at 6 different time points; 0 min (blue), 15 min (red), 30 min (gray), 2 h (yellow), 5 h (dark blue) and 8 h (green) for the ANS data and 10 h (green) for the NR data. Fluorescence intensity of fluorophore with 1: Buffer only, 2: Particle only, 3: Lysozyme + particle, 4: HSA + particle, 5: OVA + particle, 6: CA-I + particle, 7: trCA-II + particle, 8: β-lactoglobulin + particle, 9: Calbindin + particle. Sky blue lines show the protein controls (i.e. protein with fluorophore) I_F_ at each time point. In both panels a yellow dashed line is drawn across all samples representing the mean fluorescence from the particle control at time point 0 min. In panel A, two insets are also shown for trCA-II (number 7) and for β-lactoglobulin(number 8) data.

**Fig 4:**
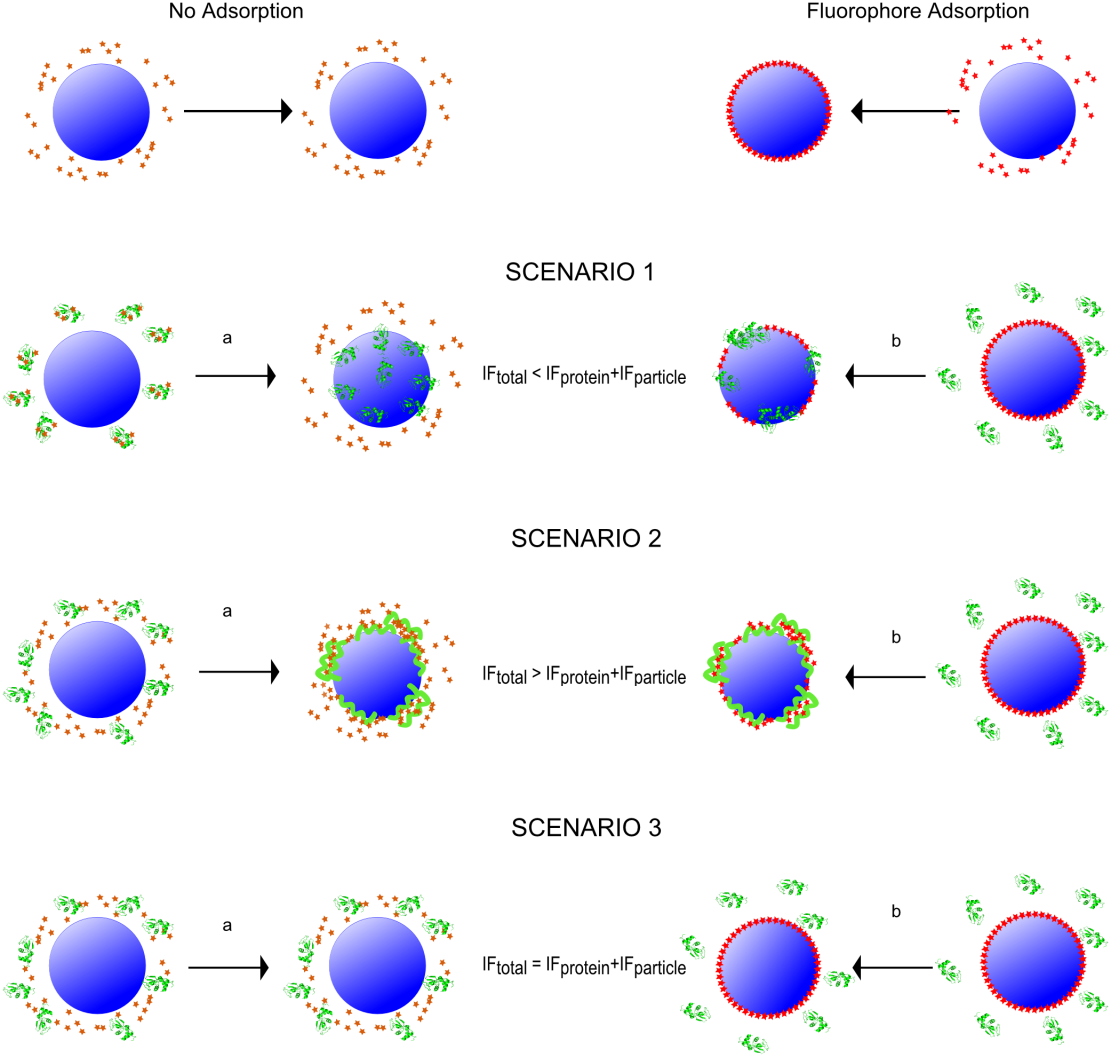
Schematic illustration of the 6 scenarios leading to the observed fluorescence intensity. Left side shows the situation when the fluorophore **do not** adsorb to the nanoparticle (1a, 2a, and 3a) and the right side shows the situations when the fluorophore **do** adsorb to the nanoparticles (1b, 2b, and 3b).

The feasibility of the screening method is demonstrated using seven different proteins; human serum albumin (HSA), chicken egg lysozyme (Lysozyme), chicken egg albumin (OVA), bovine β-lactoglobulin (β-lactoglobulin), human carbonic anhydrase I (CA-I), human carbonic anhydrase II truncated at position 17 (trCA-II) and bovine Calbindin D9k (Calbindin), two different nanoparticles (carboxyl- and amine-modified polystyrene nanoparticles), and two different fluorophores (ANS and NR) reporting on the same property i.e. accessible hydrophobic surfaces. The method will generate results for most metallic, metal oxide, organic and natural nanoparticles as long as the right experimental conditions are chosen. Physical properties of the chosen proteins and particles can be seen in S1 Table, and the raw data (averaged from 3 replicates) are presented in Fig 2. The fluorescence intensity is monitored at three different wavelengths (460, 475, and 520 nm) that span the emission spectrum of ANS, to ensure capturing a significant I_F_ change. Results for the same experiment, with NR as fluorophore, can be seen in S1 Fig. Due to the diverse contributions to the signal in the raw data, a detailed data analysis is required in order to draw conclusions. Fig 3 shows the data for the interactions of carboxylated polystyrene nanoparticles (PS-COOH) with proteins. Each bar represents the mean I_F_ value of three replicates, with standard deviation, for each PS-COOH and protein pair at different time points. The fluorescence intensity change can either be positive, negative or zero compared to the sum of controls. The output from the screening can be related to six most likely scenarios for the interplay of components in a protein—nanoparticle system which are illustrated in Fig 4. Depending on whether the fluorophore adsorbs to the investigated particle or not, the scenarios can be divided into two different blocks.

No fluorophore adsorption to the particle (block a).

1a: The protein adsorbs to the particles and consequently fluorophore molecules that were bound to the protein are released into the bulk. The I_F_ is lower in the protein—nanoparticle sample compared to the sum of the nanoparticle and protein controls.
2a: The protein adsorbs to the nanoparticles and undergoes structural rearrangements leading to exposure of hydrophobic patches to which the fluorophore can bind. The I_F_ is higher in the protein—nanoparticle sample compare to the sum of the nanoparticle and protein controls.
3a: The protein does not adsorb to the particles or the protein-nanoparticle complex does not expose any further hydrophobic surface to which the fluorophore can bind. No difference in I_F_ can be observed between the protein—nanoparticle sample and the sum of controls.

The fluorophore adsorbs to the particle (block b).

1b: The protein has higher affinity for the particle surface than the fluorophore has and displaces the fluorophore from the particle surface. The I_F_ is lower in the protein—nanoparticle sample compared to the sum of the protein and particle controls.
2b: Protein and fluorophore adsorb on the particle surface. Additionally, hydrophobic patches of the protein are exposed to the bulk, to which fluorophore molecules can bind. The I_F_ for the protein—nanoparticle sample is higher than the sum of the protein and particle controls.
3b: The protein has lower affinity for the particle surface than the fluorophore, or the formed complex has the same I_F_ as the summed controls. The I_F_ for the protein—nanoparticle sample is equal to the sum of the protein and particle controls.

Positive interaction hits are given only by scenarios 1a, 1b, 2a, and 2b. The method is designed for high throughput screening and additional experiments are strongly encouraged to validate a specific observation (for example isothermal calorimetry, analytical ultracentrifugation, circular dichroism, intrinsic flourescence etc). This pertains both to positive (scenario 1 and 2) and negative (scenario 3) hits. As an example, results that indicate no adsorption of a specific protein on a specific particle has to be confirmed with another complementary method since a negative result does not necessarily imply absence of interactions. However, depending on the aim of the investigation, the screening results will clearly indicate for which systems a more thorough investigation is necessary to reveal the mode of interaction.

The present method is designed for high throughput; however, there will be situations for which other approaches may be more suitable. For example, instead of using fluorophores in bulk one can chose to specifically label the protein with a fluorophore, specifically label the particle surface with a fluorophore or label the protein with stable isotopes. However, also these mentioned methods have their own drawbacks and require more steps, compromising the high throughput advantage of the method described here.

## Summary of Screening Data for PS-COOH Particles

The different scenarios described above can be used for analyzing the results presented in Fig 3 which shows the time evolution of the I_F_ for the combined protein-nanoparticle (PS-COOH) samples as well as the controls monitored with ANS and NR. The data indicate that ANS does not adsorb to the PS-COOH particles since there is no difference between I_F_ of ANS in buffer and I_F_ of ANS with particles (see also S2 Fig for nanoparticle:ANS titration results). Given those evidences, scenarios 1a, 2a and 3a are possible for the combined systems monitored by ANS. Similarly, Fig 3B shows that NR readily adsorbs to the PS-COOH particles, as demonstrated by the titration results in S2 Fig, which means that scenarios 1b, 2b and 3b are valid when NR is used. With this in mind, the different samples have been classified according to the six different scenarios.

Considering the classification obtained by the analysis of the screening experiment and DLS data (see Table 1) on the protein-nanoparticle system, the following conclusions can be drawn:

**Table 1:** NP sizes before and after screening experiment.

	PS-COOH + ANS^1^	PS-COOH + NR^2^	PS-NH_2_ + ANS^1^	PS-NH_2_ + NR^2^
	Size (nm) ± Std dev	Size (nm) ± Std dev	Size (nm) ± Std dev	Size (nm) ± Std dev
In Hepes buffer	46.1 ± 0.3	46.1 ± 0.3	52.7± 0.7	52.7 ± 0.7
+ Fluorophore	51.1 ± 1.3	47.6 ± 0.7	Aggregated	57.3 ± 1.0
With protein:				
Lysozyme	V.A.^3^	V.A.^3^	V.A.^3^	51 ± 6.1
HSA	52.3 ± 1.0	50.4 ± 0.5	~64 and 250	V.A.^3^
OVA	50.2± 0.8	50.6 ± 0.6	~90 and 300	V.A.^3^
CA-I	52.3± 1.0	48.5 ± 1.4	V.A.^3^	58.8 ± 1.6
trCA-II	52.5± 0.5	50.5 ± 1.6	V.A.^3^	56.1 ± 3.8
β-Lactoglobulin	53.3± 0.9	48.9 ± 1.6	144.5± 5.1	V.A.^3^
Calbindin	52.7± 0.3	49.4 ± 0.5	V.A. ^3^	V.A. ^3^

^1^ Size measured by DLS, ~48 h after sample mixture.

^2^ Size measured by DLS, ~12 h after sample mixture.

^3^ Visible precipitated aggregates

- Lysozyme can be sorted to follow scenarios 2a and 1b. The I_F_ of the Lysozyme-nanoparticle sample increases over time when ANS is used as a reporter fluorophore, however, Lysozyme alone binds ANS resulting in an increase of I_F_ over time which complicates the interpretation. A closer inspection of the data reveals that the I_F_ changes of the Lysozyme-nanoparticle sample is higher than the combined controls (Fig 3A). This indicates that Lysozyme adsorbs to the PS-COOH surface and undergoes structural changes exposing new hydrophobic patches to which ANS can bind; i.e. scenario 2a. This finding is also confirmed by a lower I_F_ value of the particle-protein system compared to the controls when monitored by NR, scenario 1b. Moreover, the particles aggregate in presence of Lysozyme as confirmed by DLS, see Table 1. This fact can arise from the unfolding of Lysozyme at the particle surface which trigger the aggregation of nanoparticle-protein complex by screening the nanoparticles surface charges.
- trCA-II also shows a significant and immediate increase of the I_F_, see inset labelled 8 in Fig 3A, indicating an instant adsorption with an immediate conformational change, scenario 2a.
- β-lactoglobulin shows an immediate significant decrease of the I_F_ in the ANS experiment, see inset labelled 7 in Fig 3A, indicating an instant adsorption to the particle surface with release of protein bound ANS as a result, scenario 1a.
- Finally, the NR data indicate that Calbindin interacts with PS-COOH-NR complex leading to a higher I_F_ than the combined controls, scenario 2b.

The other proteins fall into scenarios that suggest no interaction between protein and PS-COOH. However, a negative hit must always be confirmed with alternative methods, as stated earlier.

## Summary of Screening Data for PS-NH_2_ Particles

**Fig 5:**
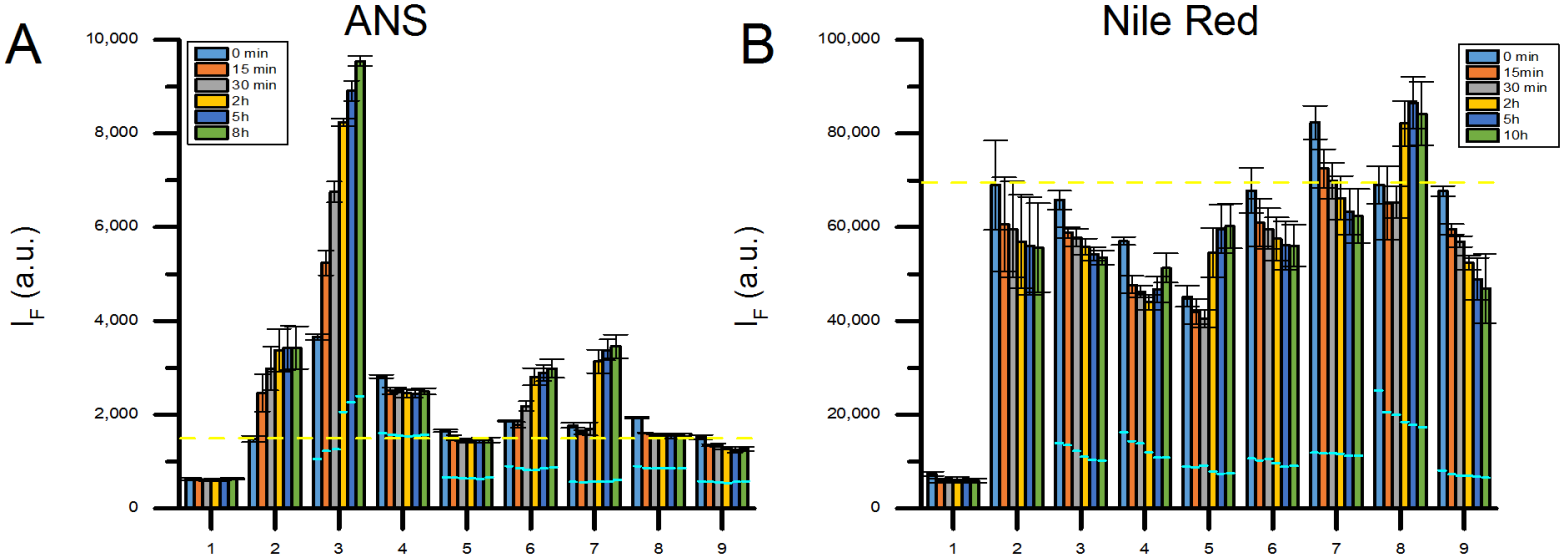
Screening results for 7 proteins and PS-NH_2_, ANS data in panel A and NR data in panel B, with corresponding controls. Bars represent the mean intensity value from three individual samples, with standard deviation in corresponding error bars, at 6 different time points; 0 min (blue), 15 min (red), 30 min (gray), 2 h (yellow), 5 h (dark blue) and 8 h (green) for the ANS data and 10 h (green) for the NR data. Fluorescence intensity of fluorophore with 1: Buffer only, 2: Particle only, 3: Lysozyme + particle, 4: HSA + particle, 5: OVA + particle, 6: CA-I + particle, 7: trCA-II + particle, 8: β-lactoglobulin + particle, 9: Calbindin + particle. Sky blue lines show the protein controls (i.e. protein with fluorophore) I_F_ at each time point. In both panels a yellow dashed line is drawn across all samples representing the mean fluorescence from the particle control at time point 0 min.

Similar analysis can be done for the amine-modified polystyrene (PS-NH_2_) nanoparticle-protein systems, see Fig 5. However, PS-NH_2_ is not colloidally stable and aggregates in presence of ANS as strongly supported by DLS experiments (Table 1), presumably due to the screening of electrostatic interactions that keeps the colloidal solution stable. Because the particles aggregate into micron size aggregates and fall out of the solution, the results cannot be evaluated according to any of the described scenarios above. However, the data still reveal interesting aspects especially when evaluated in combination with a technique that measures particle (aggregate) size like DLS (see above). On the other hand, NR does not compromise the colloidal stability of the PS-NH_2_ particles (see Table 1) but adsorb to the particles as evident from the titration curves in S2 Fig. Hence, the different samples can be classified into scenarios 1b, 2b and 3b.

Combining the data from the screening experiment with two different dyes and DLS for the PS-NH_2_ particles, the following can be concluded:

- According to the ANS data, Lysozyme affects the stability of PS-NH_2_–ANS and accelerates the co-precipitation of the whole system, eventually leading to higher I_F_ than the sum of protein and particle controls. The co-precipitation process goes on throughout the experiment. On the other hand, NR data show that Lysozyme falls within the standard deviation of the particle control I_F_, and DLS data show that the sample is colloidal stable at nominal size. The last described findings indicate no or weak interaction (Scenario 3b) between Lysozyme and PS-NH_2_.
- Data for HSA, OVA and β-lactoglobulin are more complex. At early time points in the NR experiment, the I_F_ of the protein-particle sample is lower than the sum of the controls i.e. it implies scenario 1b, hence, these proteins compete with NR to adsorb to the particle surface. However, with longer incubation times an increase in the I_F_ can be observed, representing major structural rearrangements, aggregation or both. DLS data (see Table 1) obtained 12 hours after sample mixing or later, indicate that the HSA, OVA, and β-lactoglobulin samples contain visible aggregates, however, this does not rule out the possibility that the proteins undergo structural rearrangements at the nanoparticle surface. The ANS data further strengthen the observation that HSA, OVA, and β-lactoglobulin adsorbs to PS-NH_2_. As shown in Fig 5, the I_F_ is constant over time compared to the particle control. Hence, these proteins interact with and stabilize the nanoparticle suspension. The DLS data, Table 1, confirm this observation. No precipitation of aggregated material is observed in contrast to the particle control. However, aggregates are still formed with sizes significantly larger than the nominal nanoparticle size. This means that protein-particle systems are more colloidal stable than the particle control. Hence, these set of proteins screen the ANS—NP interaction by competing for the nanoparticles surface. This could be further explored by changing the sample mixing order and adding the fluorophore last but that is beyond the scope of the article.
- Calbindin shows similar results as HSA, OVA and β-lactoglobulin, however, it lacks the I_F_ increase in the NR data and does not distinguish itself from the particle control in the NR experiment. The Calbindin-nanoparticle sample generated large aggregates out of the detection range of the DLS instrument but they did not precipitate. Hence, it needs to be further investigated.
- CA-I and trCA-II shows opposite trends for the NR, ANS and DLS experiments compared with HSA, OVA and β-lactoglobulin. NR shows that CA-I and trCA-II most likely do not adsorb to the PS-NH_2_ particles, they fall into scenario 3b. DLS measurements of the NR sample also show that the particles are colloidally stable and the measured sizes do not differ or deviate from the particle control. On the other hand, the ANS data shows that CA-I and trCA-II only initially stabilize the particle which aggregates afterwards, indicating a transient interaction between the proteins and the nanoparticle, see Fig 5 and Table 1. This transient interaction is then replaced by the ANS adsorption that eventually destabilizes the system and triggers the precipitation of the colloidal particles.

According to the NR data for the PS-NH_2_ systems; Lysozyme, CA-I, trCA-II, and Calbindin all falls into scenario 3b, hence, this should in principle account for no interaction. However as indicated previously a negative hit cannot be interpreted as a lack of interaction. In fact the ANS data suggest that some of those proteins interact either with particles or particle-flourophore complexes (see above). Inspection of the NR data also reveals that the standard deviation for the particle control is quite large compared to most of the protein-particle samples indicating that the proteins contribute to stabilizing the mixture.

## Screening Results Compared to Published Data

The conclusions regarding interactions from the screening of the seven different proteins with two different surface modified polystyrene nanoparticles are summarized in Table 2. This data set demonstrates many of the possibilities and some of the limitations of the method. For some of the systems used to benchmark the method for screening of interactions between proteins and nanoparticles the results obtained can be compared to previously published data.

**Table 2:** Summary of the screening data.

	PS-COOH			PS-NH2		
	+ ANS	+ NR		+ ANS	+ NR	
Protein	Sce.^a^	Sce.^a^	C. A.^b^	Sce.^a^	Sce.^a^	C. A.^b^
Lysozyme	2a	1b	Ads^c^	n/a^d^	3b	-
HSA	3a	3b	-	n/a^d^	1b	Ads^c^
OVA	3a	3b	-	n/a^d^	1b	Ads^c^
CA-I	3a	3b	-	n/a^d^	3b	-
trCA-II	2a	3b	Ads^c^	n/a^d^	3b	-
β-lactoglobulin	1a	3b	Ads^c^	n/a^d^	1b	Ads^c^
Calbindin	3a	2b	Ads^c^	n/a^d^	3b	-

^a^ Scenario No. as presented in the text and Fig 4.

^b^ Combined Analysis taken in consideration also the DLS data in Table 1.

^c^ The protein adsorbs to the nanoparticle.

^d^ not applicable.

Cukalevski et al. has reported interactions between Lysozyme and HSA with PS-COOH (24 nm diameter) and PS-NH_2_, by circular dichroism and intrinsic tryptophan fluorescence [10]. Even though there are differences in experimental conditions (i.e. sample buffer and PS-COOH particle size), some general comparisons can be made. Both our ANS and NR data, show that Lysozyme adsorbs to the PS-COOH and the ANS data indicate that Lysozyme undergoes structural changes which is also in line with Cukalevski et al. findings [10]. To further evaluate the screening method an experiment was set up to study the effect of conducting the screening in PBS compared to HEPES for Lysozyme and HSA, see S3 Fig. In the case of Lysozyme and PS-COOH nanoparticles it is evident that the buffer difference does not significantly change the trends; Lysozyme undergoes structural rearrangement in both buffers. When it comes to Lysozyme and PS-NH_2_, the NR experiment indicate that there are no or very limited interaction between the protein and particle which is along the results that Cukalevski et al. has reported [10].

Both ANS and NR show that a prospective interaction between HSA and PS-COOH that does not cause any major structural changes in the protein which is in line with what Cukalevski et al. showed before [10]. However, the screening cannot detect the interaction (not even in PBS, see S3 Fig) that could be responsible for the decrease of intrinsic fluorescence that Cukalevski et al. showed. Whether the differences arise from the different size of PS-COOH particles used or the reporting properties of the fluorophore has to be further investigated. trCA-II do adsorb to PS-COOH while CA-I do not, according to the screening results. This is compatible with ITC results presented by Assarsson et al. [37].

## Conclusion

In summary, the presented method is flexible and can readily be adjusted to different conditions. It is high throughput and generates a significant amount of data as many protein—particle combinations can be quickly explored in parallel and even at different emission wavelengths since a multi well plate is used instead of a cuvette. Moreover, interactions and structural changes that occur on a time scale from milliseconds (for plate readers equipped with an injector) to days can be monitored. The method takes advantage of the high sensitivity of fluorescence spectroscopy together with the diverse selection of fluorophores that could be used. Moreover, different protein-nanoparticle interaction properties can be monitored by using different property-sensitive dyes. Furthermore, low sample amount is required and therefore considerable amount of data is obtained at lower cost. Finally, the method also generates information about the colloidal stability of particles.

## Materials

### Plate reader

Plate reader measurements were done in BMG FLUOstar for ANS measurements and CLARIOstar for NR measurements. For ANS experiments, filters were set as following: λ_ex_: 320 nm, λ_em_: 460, 475, and 520 nm. For NR experiments, monochromator range was set as following: λ_ex_: 550±10 nm, λ_em_: 600±10 nm and 660±10 nm. In all experiments, samples were excited and emission was read from the bottom, in order to avoid the interference from the formed meniscus. Any plate reader with similar capacities can be used.

### Plates

96 well half-area plate of black polystyrene with clear bottom and non-binding surface (Corning 3881) was used for all screening experiments. Any plate, with different volume and well number from a different manufacturer can be used.

### Fluorophore

8-Anilino-1-naphthalenesulfonic acid ammonium salt (ANS) were purchased from Sigma Aldrich and used without further purification. ANS was dissolved in filtered water to reach a stock concentration of 1.3 mg/ml which is diluted to a final concentration of 0.195 mg/ml in the well. NR was a kind gift from Prof. Lo Gorton. NR was dissolved in DMSO to get a stock of 100 μM. In order to limit the amount of DMSO in the sample wells, final NR concentration was set to 1 μM.

### Sample buffer

10 mM HEPES buffer pH 7.4 was used throughout all experiments except for some data shown in S3 Fig which was with PBS buffer pH 7.4.

### Proteins

Human Serum Albumin (lyophilized powder, essentially fatty acid free), Chicken Egg Lysozyme (lyophilized powder, protein ≥90%, ≥40,000 units/mg protein), Chicken Egg Albumin (lyophilized powder, protein 99%) and Bovine β-lactoglobulin (lyophilized powder, contains a and b) were purchased from Sigma Aldrich and used without further purification. Wild type Human carbonic anhydrase I was a kind gift from Prof. Bengt-Harald Jonsson. The plasmid for production of Truncated at position 17 in the N-terminal Human carbonic anhydrase II pseudo wilt type was a kind gift from Prof. Bengt-Harald Jonsson and the protein was expressed and purified according to [38]. Calbindin D_9k_ expressed and purified according to [39, 40].

All proteins were dissolved in working buffer and centrifuged at 14000 rpm for 5 minutes to get rid of big aggregates. Concentrations were determined spectrophotometrically using (a Shimadzu UV-1800 or Agilent 8453 spectrophotometer) with extinction coefficients: 37970 M^-1^.cm^-1^, 34445 M^-1^.cm^-1^, 31775 M^-1^.cm^-1^, 46800 M^-1^.cm^-1^, 44100 M^-1^.cm^-1^, 17210 M^-1^.cm^-1^ and 1490 M^-1^.cm^-1^ for Lysozyme, HSA, OVA, CA-I, trCA-II, β-lactoglobulin and Calbindin, respectively. Final concentrations for all proteins in the well were set to 0.1 mg/ml.

All measurements were conducted at 30°C.

### Particles

Particles were purchased from Bangs Laboratories, Inc. and Polyscience. All particles were dialyzed against water, diluted with buffer to a stock solution of around 2 mg/ml and the concentration checked using absorbance (measured at 270 and compared to a standard curve done beforehand using the manufacturer mass concentration of the stock solution) before use. The particle size in sample buffer was estimated using DLS; being 48 nm for the PS-COOH and 53 nm for the PS-NH_2_ particles. The mass concentration, mean size and density of the particles were used in the calculation to ensure a fixed total particle surface area in the samples (the calculation assumes that the particles are spherical and homogeneous) and the final particle concentration in the wells were 0.2 and 0.23 mg/ml for PS-COOH and PS-NH_2_ respectively. The characterization of the particles is reported in S1 Table. Prior to each experiment, the hydrodynamic diameters of NPs was checked using a DLS plate reader (DynaPro Plate Reader II, Wyatt Technology, Santa Barbara, CA) operating with a 158° scattering angle at 30°C using a clear, flat-bottom 96 well black plate (Costar). 3 replicates of each sample were measured with 10 acquisitions per sample. Each acquisition was set to 5 seconds. A general purpose analysis model (cumulant fit for monomodal dispersions and regularization fit for multimodal dispersions) was employed to determine the hydrodynamic diameter of the particles.

## Supporting Information

S1 Fig NR Screening raw data generated by the plate reader. The interactions between 7 proteins and 2 particles was followed with NR fluorescence monitored at 2 emission wavelength; black = 600 nm and gray = 660 nm, over time. The showed results are the average of three sample replicates.
(TIF)

S2 Fig Change of fluorescence intensity upon titration of nanoparticles into 1 μM fluorophore. The graphs shows the results from titration experiments which were conducted to further investigate how the fluorophores interacts with the nanoparticles. The I_F_ does not change significantly for the ANS:PS-COOH system, which is a clear indication that ANS does not adsorb to the PS-COOH surface. For the other 3 combination a clear change in I_F_ are observed, which is a clear indication that the fluorophores adsorbs to the particle surface. Each data point represents the average of three measurements and the error bars shows the standard deviation.
(TIF)

S3 Fig Processed screening results for Lysozyme + PS-COOH and HSA + PS-COOH systems with ANS as reporter fluorophore for two different buffer systems; PBS and HEPES. Bars represent the mean intensity value from three individual samples, with corresponding error bars, at 6 different time points; 0 min (blue), 15 min (red), 30 min (gray), 2 h (yellow), 5 h (dark blue) and 8 h (green).
(TIF)

S1 Table Particle Properties and Protein Molecular Weights, Theoretical pIs and ExPASy Accession Numbers. (DOCX)
