# Supplementary figures and images for: High Throughput Screening Method to Explore Protein Interactions with Nanoparticles
Source: PLoS One. 2015 Aug 27;10(8):e0136687. doi: 10.1371/journal.pone.0136687 (PMC4551901; doi:10.1371/journal.pone.0136687)

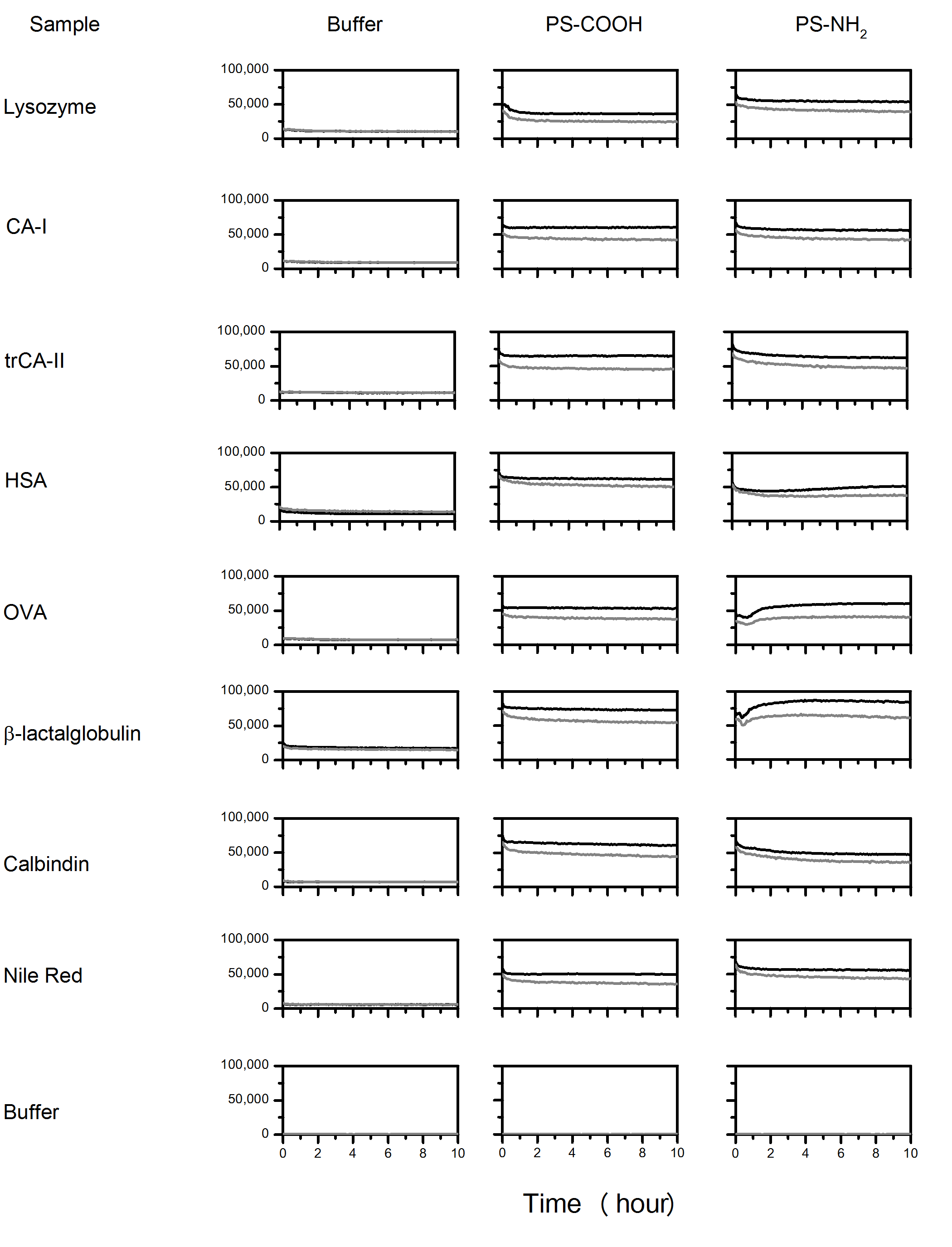

Supplement: S1 Fig — The interactions between 7 proteins and 2 particles was followed with NR fluorescence monitored at 2 emission wavelength; black = 600 nm and gray = 660 nm, over time. The showed results are the average of three sample replicates. (TIF) [file pone.0136687.s001.tif]

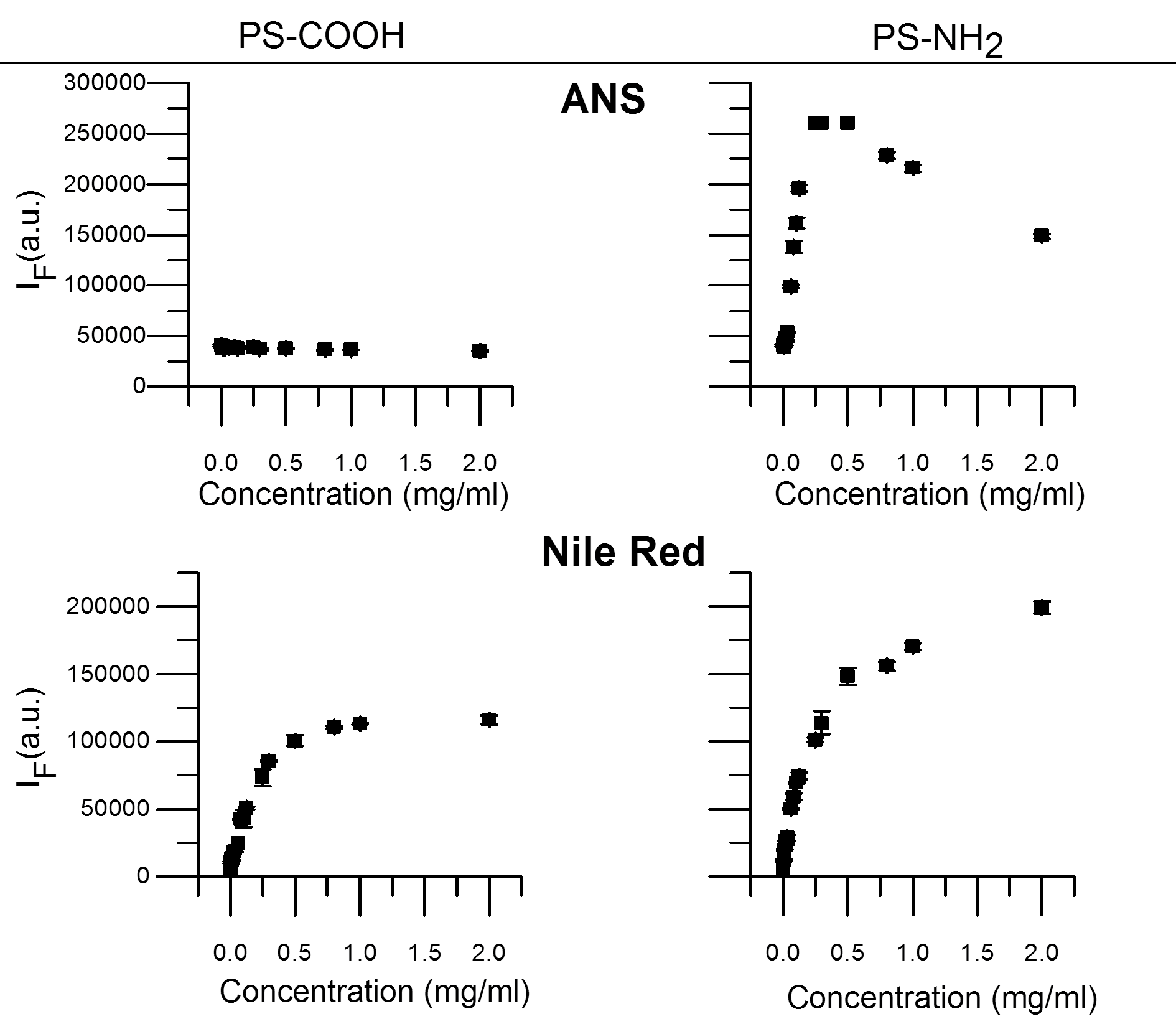

Supplement: S2 Fig — The graphs shows the results from titration experiments which were conducted to further investigate how the fluorophores interacts with the nanoparticles. The IF does not change significantly for the ANS:PS-COOH system, which is a clear indication that ANS does not adsorb to the PS-COOH surface. For the other 3 combination a clear change in IF are observed, which is a clear indication that the fluorophores adsorbs to the particle surface. Each data point represents the average of three measurements and the error bars shows the standard deviation. (TIF) [file pone.0136687.s002.tif]

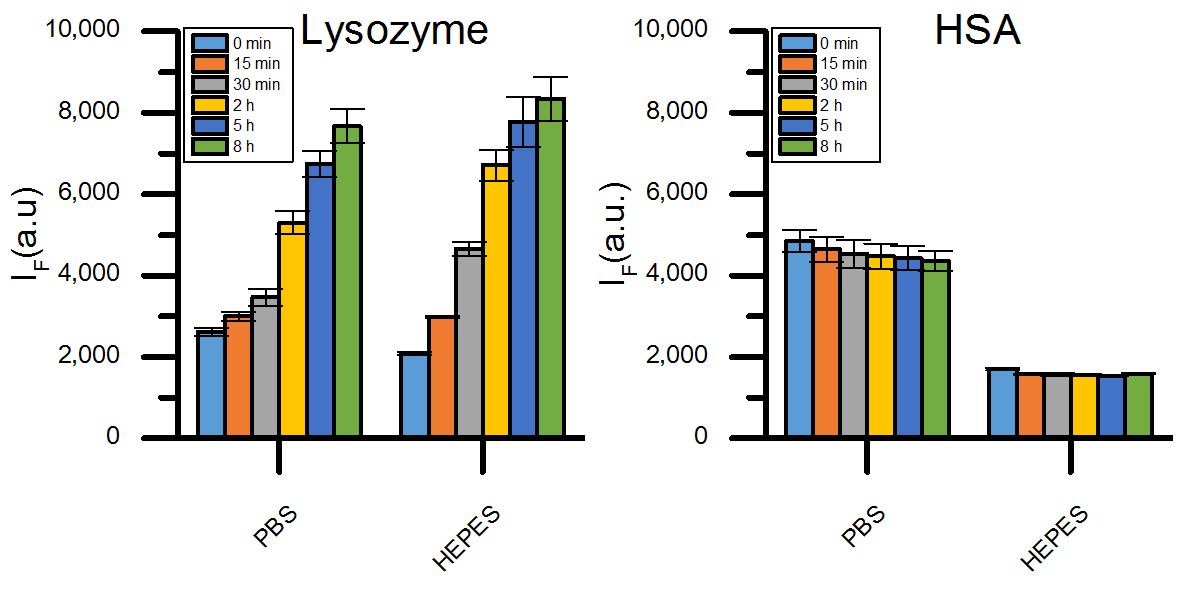

Supplement: S3 Fig — Bars represent the mean intensity value from three individual samples, with corresponding error bars, at 6 different time points; 0 min (blue), 15 min (red), 30 min (gray), 2 h (yellow), 5 h (dark blue) and 8 h (green). (TIF) [file pone.0136687.s003.tif]
