# Supplementary material for: High Throughput Screening Method to Explore Protein Interactions with Nanoparticles
Source: PLoS One. 2015 Aug 27;10(8):e0136687. doi: 10.1371/journal.pone.0136687 (PMC4551901; doi:10.1371/journal.pone.0136687)
Supplement: S1 Table — (DOCX) [file pone.0136687.s004.docx]

**S1** **Table. Particle Properties and Protein Molecular Weights, Theoretical pIs and ExPASy Accession Numbers.**

| Protein | Accession Number | Mw (g/mol) | Theoretical pI^1^ |
| --- | --- | --- | --- |
| Chicken Lysozyme | P00698 | 14313 | 9,3 |
| Human Serum Albumin | P02768 | 66472 | 5,7 |
| Chicken Egg Albumin | P01012 | 42881 | 5,2 |
| Human Carbonic Anhydrase I | P00915 | 28739 | 6,6 |
| Trunc17 Human Carbonic Anhydrase II | n.a. | 27170 | 6,7 |
| Bovine β-lactoglobulin | P02754 | 18281 | 4,8 |
| Human Calbindin D9k | P29377 | 8886 | 4,7 |

| Particle | Manufacturer | Size (nm)^2^ | Size (nm)^3^ | Z-pot. (mV)^3^ | Density (g/ml)^4^ |
| --- | --- | --- | --- | --- | --- |
| PS-NH_2_ | Polysciences, Inc. | 58 | 53±1 | 42±2 | 1.052 |
| PS-COOH | Bangs Lab. | 43 | 46±1 | -44±4 | 1.052 |

^1^ Calculated with ProtParam at: Http://web.expasy.org

^2^ According to manufacturer.

^3^ Measured after the particles had been dialyzed against water and diluted in Hepes buffer.

^4^ According to manufacturer.
